# Modulation of kanamycin B and kanamycin A biosynthesis in *Streptomyces kanamyceticus* via metabolic engineering

**DOI:** 10.1371/journal.pone.0181971

**Published:** 2017-07-28

**Authors:** Wenli Gao, Zheng Wu, Junyang Sun, Xianpu Ni, Huanzhang Xia

**Affiliations:** School of Life Science and Biopharmaceutics, Shenyang Pharmaceutical University, No.103 Wenhua Road, Shenyang, Liaoning, China; Universite Paris-Sud, FRANCE

## Abstract

Both kanamycin A and kanamycin B, antibiotic components produced by *Streptomyces kanamyceticus*, have medical value. Two different pathways for kanamycin biosynthesis have been reported by two research groups. In this study, to obtain an optimal kanamycin A-producing strain and a kanamycin B-high-yield strain, we first examined the native kanamycin biosynthetic pathway *in vivo*. Based on the proposed parallel biosynthetic pathway, *kanN* disruption should lead to kanamycin A accumulation; however, the *kanN*-disruption strain produced neither kanamycin A nor kanamycin B. We then tested the function of *kanJ* and *kanK*. The main metabolite of the *kanJ*-disruption strain was identified as kanamycin B. These results clarified that kanamycin biosynthesis does not proceed through the parallel pathway and that synthesis of kanamycin A from kanamycin B is catalyzed by KanJ and KanK in *S*. *kanamyceticus*. As expected, the kanamycin B yield of the *kanJ*-disruption strain was 3268±255 μg/mL, 12-fold higher than that of the original strain. To improve the purity of kanamycin A and reduce the yield of kanamycin B in the fermentation broth, four different *kanJ*- and *kanK*-overexpressing strains were constructed through either homologous recombination or site-specific integration. The overexpressing strain containing three copies of *kanJ* and *kanK* in its genome exhibited the lowest kanamycin B yield (128±20 μg/mL), which was 54% lower than that of the original strain. Our experimental results demonstrate that kanamycin A is derived from KanJ-and-KanK-catalyzed conversion of kanamycin B in *S*. *kanamyceticus*. Moreover, based on the clarified biosynthetic pathway, we obtained a kanamycin B-high-yield strain and an optimized kanamycin A-producing strain with minimal byproduct.

## Introduction

The secondary metabolites of *Streptomyces* are rich and diverse, and some of these metabolites are complex and obtained at low yields, which makes their extraction and purification difficult. Efforts to improve *Streptomyces* strains are aimed at obtaining overproducing cultures and developing new strains that can produce the desired products as the major components. Metabolic engineering is a widely accepted method that can improve a strain in a targeted manner [1] and is facilitated by gene function and biosynthetic pathway information.

**Fig 1:**
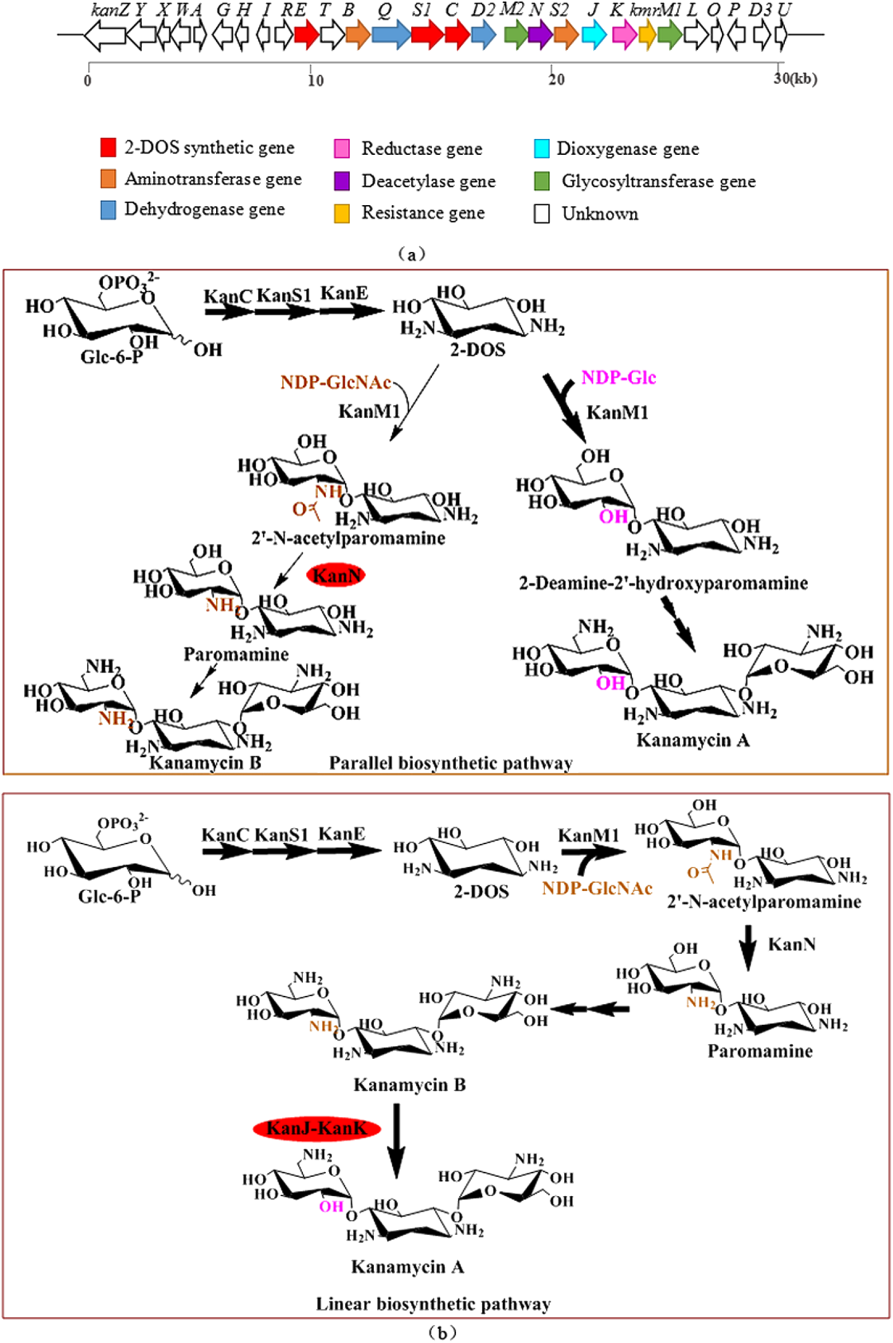
Kanamycin biosynthetic gene cluster and proposed kanamycin biosynthetic pathways. **(a)** Kanamycin biosynthetic gene cluster. **(b)** Proposed parallel and linear kanamycin biosynthetic pathways.

Kanamycin is an effective aminoglycoside antibiotic produced by *Streptomyces kanamyceticus*. Kanamycin A is the main product, and kanamycin B as the corresponding byproduct (5%~7% of total production). Kanamycin A has a hydroxy group at position C2′, whereas kanamycin B possesses an amino group at this position (Fig 1B). Their specific interactions with bacterial ribosomal RNAs to inhibit bacterial protein synthesis have been clearly observed through X-ray structural analysis [2].

Kanamycin B is a precursor for semisynthetic antibiotics such as arbekacin and dibekacin [3–5] and is generally extracted from the broth of *S*. *kanamyceticus*. However, the yield of kanamycin B is too low for industrial applications, necessitating strain improvements to maximize kanamycin B production [6,7].

Due to differences in their C2′ substituents, kanamycin B is less biologically active [8] and has stronger side effects [9–11] than kanamycin A. According to the European Pharmacopoeia, kanamycin B must be present at less than 4% in kanamycin monosulfate and kanamycin acid sulfate [12]. Therefore, kanamycin B, as an impurity, must be minimized to engineer an optimal kanamycin A-producing strain.

Studies of kanamycin biosynthetic pathways provide information and direction for strain improvement. The kanamycin biosynthetic gene cluster was first isolated from *S*. *kanamyceticus* in 2004 [8] (GenBank ID: AJ628422). Kharel et al. [13] also cloned a sequence from *S*. *kanamyceticus* that proved to be a kanamycin biosynthesis gene cluster containing 40 ORFs (GenBank ID: AB164642). The synthetic routes of kanamycin, as an aminoglycoside antibiotic, are very similar to those of other aminoglycoside antibiotics, and the homology among their functionally similar genes is relatively high [14]. In fact, genes that encode enzymes that synthesize 2-DOS are highly conserved in the biosynthetic gene clusters of aminoglycoside antibiotics.

Most studies of the kanamycin biosynthetic gene cluster have employed homologous comparison and heterologous expression. Nepal et al. [15] expressed *kanN*, *kanS1*, *kanE*, *kanM1*, and *kanN* in *Streptomyces lividans*, and their results confirmed that these genes are involved in paromamine biosynthesis (Fig 1A). According to heterologous expression and biochemical experiments, two different kanamycin biosynthetic pathways have been proposed by different research groups. In 2011, through heterologous expression of kanamycin biosynthetic genes in *Streptomyces venerzuelae*, Park et al. [6] demonstrated that kanamycin A and kanamycin B are synthesized by two parallel pathways due to the substrate promiscuity of the first glycosyltransferase. Kanamycin A is the main product because the optimal substrate for the glycosyltransferase is NDP-Glc; kanamycin B is a byproduct because glycosyltransferase can also utilize a small amount of NDP-GlcNAc (Fig 1B). However, in 2012, Sucipto and colleagues showed that KanJ and KanK transform kanamycin B to kanamycin A. A bioinformatics analysis of the kanamycin gene cluster revealed that it encodes the following two unique enzymes: an α-ketoglutarate-dependent non-heme iron dioxygenase, KanJ and an NADPH-dependent reductase, KanK. Sucipto et al. [16] expressed KanJ and KanK in *E*. *coli*, and their *in vitro* catalytic experiments demonstrated that kanamycin B is first converted to 2′-oxokanamycin through a reaction catalyzed by KanJ and that 2′-oxokanamycin is then selectively reduced by KanK in the presence of NADPH to yield kanamycin A. In short, researchers have proposed both parallel and linear biosynthetic pathways (Fig 1B), but the biosynthetic pathway that operates in *S*. *kanamyceticus* remains unclear.

The original strain *S*. *kanamyceticus* CG305 used in this study was selected from *S*. *kanamyceticus* CGMCC 4.1441 due to its higher levels of kanamycin biosynthesis. *S*. *kanamyceticus* CG305 was obtained via multiple UV and chemical mutagenesis, without any operation of genetic engineering. To obtain a kanamycin B-high-yield strain and an optimal kanamycin A-producing strain, it is essential to identify the explicit kanamycin biosynthetic pathway *in vivo*. Therefore, we first tested the kanamycin biosynthetic pathway. According to the parallel biosynthetic pathway, the 2'-*N*-acetylparomamine deacetylase *kanN* only plays a role in kanamycin B biosynthesis, and *kanN* disruption should lead to kanamycin A accumulation. A strain producing kanamycin A as a single component can thus be obtained. In contrast, if *in vivo* biosynthesis of kanamycin A is catalyzed by KanJ and KanK, disrupting *kanJ* in the original strain will result in accumulation of kanamycin B. Additionally, to improve the purity of kanamycin A and reduce the yield of kanamycin B in the fermentation product, *kanJ*-and *kanK*-overexpressing strains were constructed.

## Materials and methods

### Bacterial strains, plasmids, and growth conditions

The strains used in this work are listed in Table 1. *E*. *coli* DH5α was used as a cloning host and grown on Luria-Bertani liquid or solid medium supplemented with antibiotics when needed. Liquid CP was employed for the vegetative growth of *S*. *kanamyceticus*, and MS medium was used for the transformation and selection of *S*. *kanamyceticus* [17]. The medium used for *S*. *kanamyceticus* sporulation contained 15 g/L soluble starch, 4 g/L yeast extract, 0.5 g/L K_2_HPO_4_, 0.5 g/L MgSO_4_·7H_2_O, and 1.5 g/L agar. The seed medium contained 20 g/L soluble starch, 25 g/L cold-pressed soybean meal, 5 g/L glucose, 1.5 g/L NaNO_3_, 1 g/L yeast powder, and 1 g/L CaCO_3_. After incubation at 28°C for 40 h, 3 mL (10% (vol/vol)) of seed culture was inoculated into fermentation medium, and the resulting culture was incubated for five days. The fermentation culture medium contained 25 g/L soluble starch, 30 g/L soya bean, 60 g/L maltose, 8 g/L NaNO_3_, 0.1 g/L ZnSO_3_, and 0.07 g/L FeSO_4_.

**Table 1:** Bacterial strains used in this study.

Strain	Characteristics	Source or reference
*Escherichia coli* DH5α	Cloning host	Invitrogen
*Escherichia coli* ET12567 (pUZ8002)	Methylation-defective strain used in *E*. *coli*-*Streptomyces*	[18]
*S*. *kanamyceticus* CG305	Original strain, producer of kanamycin A and kanamycin B	This lab
*S*. *kanamyceticus* Δ*kanN*	*S*. *kanamyceticus* CG305 in which *kanN* is disrupted	This study
*S*. *kanamyceticus* Δ*kanJ*	*S*. *kanamyceticus* CG305 in which *kanJ* is disrupted	This study
*S*. *kanamyceticus* JKE1	*S*. *kanamyceticus* CG305 overexpressing *kanJ* and *kanK* by site-specific integration	This study
*S*. *kanamyceticus* JKE2	*S*. *kanamyceticus* CG305 overexpressing *kanJ* and *kanK* with homologous arms R1 and R3	This study
*S*. *kanamyceticus* JKE3	*S*. *kanamyceticus* CG305 overexpressing *kanJ* and *kanK* with homologous arms R5 and R7	This study
*S*. *kanamyceticus* JKE4	*S*. *kanamyceticus* JKE2 overexpressing *kanJ* and *kanK* with homologous arms R5 and R7.	This study

#### Construction of the *kanN*-disruption strain

The *kanN* gene was disrupted via pD2925-mediated double-crossover recombination. The plasmids and primers used in this study are listed in Table 2 and S1 Table. Standard procedures for DNA manipulation were used to construct the plasmids [19]. The plasmid pDLA302 containing *kanN* with an in-frame deletion was constructed as follows. The N1 (1747 bp) and N2 (1822 bp) fragments, representing the upstream and downstream homologous arms, respectively, were amplified by polymerase chain reaction (PCR) from *S*. *kanamyceticus* CG305 genomic DNA using primers AP1III/ AP2III and AP3III/ AP4III, respectively (S1 Fig). N1 and N2 were cloned separately into pUC18 (TaKaRa, Japan) and then excised from the resulting plasmids using *Xba*I-*EcoR*I and *Eco*RI-*Kpn*I, respectively. The excised products containing the upstream and downstream fragments of *kanN* were then ligated with the *Xba*I-*Kpn*I fragment of pD2925 to yield pDLA302 for in-frame deletion of *kanN* (S1 Fig).

**Table 2:** Plasmids used in this study.

Plasmid	Characteristics	Source
pIJ2925	Cloning vector for *E*. *coli*, *ori*(pUC18), Amp^R^	[20]
pD2925	*E*. *coli*-*Streptomyces* shuttle vector, *oriT*(RP4), *ori*(pUC18), Amp^R^, Am^R^	[21]
pSPU241	Cloning vector for *E*. *coli*, PermE^*^ promoter, *To* terminator, Amp^R^	[21]
pEAP1	*E*. *coli*-*Streptomyces* shuttle vector, *oriT*(RP4), *ori*(pUC18), *int-attP*(φC31), Amp^R^, Erm^R^	[21]
pDLA302	pD2925 containing downstream and upstream fragments of *kanN*, Amp^R^	This study
pLDJ202	pD2925 containing downstream and upstream fragments of *kanJ*, Amp^R^	This study
pHJK1	pEAP1 containing intact *PhrdB*, *kanJ* and *kanK* fragments, Amp^R^	This study
pHJK02	pD2925 containing intact *PhrdB*, *kanJ* and *kanK* fragments, R1, R3, Amp^R^	This study
pHJK03	pD2925 containing intact *PhrdB*, *kanJ* and *kanK* fragments, R5, R7, Amp^R^	This study

First, the disruption plasmid pDLA302 was introduced into *E*. *coli* ET12567/pUZ8002 via the CaCl_2_ method and then into *S*. *kanamyceticus* CG305 by conjugational transfer [17]. After incubation at 37°C for 23 h, each dish was overlaid with 1 mL of sterile water containing apramycin at a final concentration of 20 μg/mL. Because pDLA302 contains an apramycin-resistance gene, the transconjugants were selected as follows: the apramycin-resistant (for the first crossover event) phenotype was first selected, and the apramycin-sensitive (the second crossover event) phenotype was then selected to isolate *kanN*-disruption strain. The transconjugants were subsequently incubated at 37°C for seven days to select for homologous recombinants (for the first crossover event). The transconjugants were cultured on antibiotic-free medium for sporulation, and the cycle was repeated three times to enhance the probability of recombination. Single clones were replica-plated onto the apramycin-containing plates, as well as on plates without antibiotic for the sporulation. The apramycin-sensitive strains were selected based on the growth conditions of the clones on the two different plates (the second crossover event), and the expected disruption genotype was identified by PCR using the primers AY1, AY2, AY3, and AY4 (S1 Fig). To confirm the mutant, the sequence of a PCR fragment of the expected size was verified by DNA sequencing (data not shown). The selected strain was named *S*. *kanamyceticus* Δ*kanN*.

### Construction of the *kanJ*-disruption strain

The *kanJ* gene was disrupted via pD2925-mediated double-crossover recombination. The plasmid pLDJ202 containing *kanJ* with in-frame deletion was constructed as follows. The J1 (1430 bp) and J2 (1986 bp) fragments, representing the upstream and downstream homologous arms, respectively, were amplified by PCR from *S*. *kanamyceticus* CG305 genomic DNA using primers JII1/ JII2 and JII3/ JII4, respectively (S3 Fig). J1 and J2 were cloned separately into pUC18 (TaKaRa, Japan) and then excised from the resulting plasmids with *Eco*RI-*Hin*dIII and *Eco*RI-*Bam*HI, respectively. The excised products were then ligated with the *Hin*dIII-*Bam*HI fragment of pIJ2925. This plasmid was then digested with *Bgl*II, and the resulting fragment containing the upstream and downstream regions of *kanJ* was inserted into the *Bam*HI site of pD2925 to yield pLDJ202 for the in-frame deletion of *kanJ* (S3 Fig).

First, the disruption plasmid was introduced into *E*. *coli* ET12567/pUZ8002 using the CaCl_2_-based method and then into *S*. *kanamyceticus* CG305 by conjugational transfer [17]. The methods used were the same as the those described above, and the expected disruption genotype was identified by PCR using the primers JY1, JY2, JY3, and JY4 (S3 Fig). To confirm the mutant, the sequence of a PCR fragment of the expected size was verified by DNA sequencing (data not shown). The selected strain was named *S*. *kanamyceticus* Δ*kanJ*.

### Complementation of disruption mutants

The pSPU241 and pEAP1 plasmids were used to construct gene complementation vectors. The *kanN* and *kanJ* genes were amplified from chromosomal DNA from the original strain using primer pairs AE1/AE2 and JE1/JE2, respectively, and PCR products were inserted into the *Bam*HI and *Hin*dIII sites of pSPU241. The cloned genes containing the PermE* promoter and *To* terminator were digested with *Bgl*II and inserted into pEAP1 to generate pLNE02 and pLJE02. The complementation plasmids were verified by sequencing and then introduced individually into Δ*kanN* and Δ*kanJ* by conjugation. The plasmids were integrated into *S*. *kanamyceticus* chromosomal DNA via site-specific recombination. Complemented exconjugants were identified by erythromycin resistance (100 μg/mL) and confirmed by PCR (S2 and S4 Figs).

### Construction of the *kanJ-* and *kanK-*overexpressing strain through site-specific integration

The pHJK1 plasmid containing the *PhrdB* promoter, *kanJ* and *kanK* was constructed for overexpression. A fragment containing the full sequences of *kanJ* and *kanK* (2025 bp) with a 5’ *Nco*I site a 3’ *Hin*dIII site was amplified by PCR from *S*. *kanamyceticus* CG305 genomic DNA using primers JKE1/JKE2. A fragment containing the full sequence of *PhrdB* promoter (426 bp) with a 5’ *Eco*RI site and a 3’ *Nco*I site was amplified by PCR from *S*. *coelicolor* M145 genomic DNA using primers H1/H2. The resulting PCR products were cloned separately into pUC18 (TaKaRa, Japan), and the products were subsequently excised from the resulting constructs using *Nco*I-*Hin*dIII and *Eco*RI-*Nco*I and then ligated with the *Hin*dIII-*Eco*RI fragment from pSPU241. The resulting plasmid was digested with *Bgl*II, and the resulting fragment containing the full sequences of *kanJ*, *kanK* and *PhrdB* was inserted into the *Bgl*II sites of pEAP1 to generate pHJK1 (S5 Fig).

The pHJK1 plasmid was introduced into *S*. *kanamyceticus* CG305 by conjugational transfer. The plasmid was integrated into *S*. *kanamyceticus* chromosomal DNA via site-specific recombination. The expected genotype was identified by erythromycin resistance (100 μg/mL) and confirmed by PCR using primers located outside of the exchange regions (S5 Fig). Extraction of free plasmids was then performed, and no free plasmid was obtained. The overexpression strain was designated *S*. *kanamyceticus* JKE1.

### Construction of *kanJ-* and *kanK*-overexpressing strains based on homologous recombination

The pHJK02 plasmid containing upstream homologous arm R1, downstream homologous arm R3, the *PhrdB* promoter, *kanJ* and *kanK* was constructed for overexpression. R1 and R2 were amplified by PCR from *S*. *kanamyceticus* CG305 genomic DNA using primers PR1/ PR2 and PR3/ PR4, respectively, and sequentially cloned into the pIJ2925 plasmid. The R1–R3 fragment was then inserted into the suicide shuttle vector pD2925. The *Bgl*II-digested fragment of pHJK1 containing the full sequences of *PhrdB*, *kanJ* and *kanK* was inserted into the resulting plasmid, yielding pHJK02 (S6 Fig).

The pHJK03 plasmid containing upstream homologous arm R5, downstream homologous arm R7, the *PhrdB* promoter, *kanJ* and *kanK* was constructed for overexpression. R5 and R7 were amplified by PCR from *S*. *kanamyceticus* CG305 genomic DNA using primers PR5/ PR6 and PR7/ PR8, respectively, and sequentially cloned into the pIJ2925 plasmid. The R5–R7 fragment was then inserted into the suicide shuttle vector pD2925. The *Bgl*II-digested fragment of pHJK1 containing the full sequences of *PhrdB*, *kanJ* and *kanK* was inserted into the resulting plasmid, yielding pHJK03 (S7 Fig).

The pHJK02 and pHJK03 plasmids were introduced separately into *S*. *kanamyceticus* CG305 through conjugational transfer using the method that was used to construct the *kanN*-disruption strain. The expected genotype was identified by amplification of a PCR product from the genomic DNA using primers that bind outside of the exchange regions (S6 and S7 Figs). To confirm the mutant, the sequence of a PCR fragment of the expected size was verified by DNA sequencing. The overexpressing strains obtained through site-directed insertion were designated *S*. *kanamyceticus* JKE2 and *S*. *kanamyceticus* JKE3.

The pHJK03 plasmid was introduced into *S*. *kanamyceticus* JKE2 by conjugational transfer using the above-described method. The expected genotype was identified by PCR using primers that bind outside of the exchange regions (S8 Fig). To confirm the mutant, the sequence of a PCR fragment of the expected size was verified by DNA sequencing, and the overexpressing strain was designated *S*. *kanamyceticus* JKE4.

### Antibiotic isolation and analysis

Strains were cultured in 20 mL of seed medium at 28°C for 40 h. Three milliliters of (10% (vol/vol)) seed culture was then inoculated into 30 mL of fermentation medium, and the resulting culture was incubated at 28°C for five days.

The pH of the culture broth was adjusted to 2 with H_2_SO_4_, and the acidified broth was stirred for 30 min and then centrifuged (14,500 × g; 10 min). The supernatant was subsequently readjusted to pH 7 using NaOH and then re-centrifuged (14,500 × g; 10 min). The supernatant of the culture broth was prepared for bioassays and further separation and purification. A microbiological assay was performed using the *Bacillus subtilis* agar diffusion method. The supernatant was applied to strongly acidic resin 001 × 7 (Anhui Sanxing Resin Technology), and the bound substance was eluted with 2 mol/L NH_4_OH. The second cation-exchange chromatography step (Anhui Sanxing Resin Technology) was performed on weakly acidic resin (D151), and the product was eluted with a gradient of NH_4_OH (from 0.01 to 0.3 mol/L).

The eluate showing biological activity was analyzed by reversed-phase high-performance liquid chromatography (RP-HPLC) with an evaporative light scattering detector (ELSD). The kanamycin content was determined via HPLC-ELSD with a reverse C18 column with an evaporation temperature of 45°C, a nitrogen pressure of 3.5 bar, a mobile phase of 0.2 mol/L trifluoroacetic acid and a mobile flow-rate of 0.8 mL/min. Standard solutions were prepared, and peak area-concentration standard curves of kanamycin A and kanamycin B were prepared for quantitative determinations. The kanamycin A and kanamycin B levels in each sample were measured three times; to minimize error, the measurements were averaged. Each strain described herein was fermented three times to ensure that the experimental data were statistically significant. The yield of each strain represents the average of the concentration of the product in the three fermentation broths and was calibrated by the standard deviation (SD). The authentic kanamycin B and kanamycin A standards were purchased from the National Institute for the Control of Pharmaceutical and Biological Products (NICPBP).

The purified products were analyzed using an HPLC electrospray ionization mass spectrometry (ESI-MS) instrument (Bruker micro-QTOF) using the same conditions as those used in HPLC-ELSD, with the mass spectrometer set to positive mode. The ESI-MS parameters were set as follows: capillary voltage, 4500 V; nebulizer gas pressure, 0.3 bar; dry gas flow-rate, 4 L/min; and temperature, 180°C. Mass spectra were recorded in the range of 50–1000 m/z. The data were analyzed using Bruker Daltonics Data Analysis 3.4 software, and ^1^H and ^13^C nuclear magnetic resonance (NMR) data were recorded on a Bruker AV600 at a frequency of 600 MHz using D_2_O as the solvent.

To purify kanamycin B, the diameter height ratio of the resin column (001 × 7 (Anhui Sanxing Resin Technology)) used was 1:40, and the bound substance was eluted with 0.1 mol/L NH_4_OH aqueous solution at a flow rate of 0.2 mL/min. The effluent was collected by a Fraction Collector and analyzed by HPLC-ELSD. The target effluent was pooled and concentrated to determine the recovery and purity of kanamycin B.

### Assays of KanJ and KanK enzyme relative activity

The original strain, *S*. *kanamyceticus* Δ*kanJ* and *S*. *kanamyceticus* JKE1, JKE2, JKE3 and JKE4 were cultured in fermentation medium at 28°C for 72 h, and the fermentation broth was then pooled (approximately 20 mL) in a 50-mL polypropylene tube. The mycelia were recovered by centrifugation (12,000 × g; 30 min) at 0°C, and all subsequent steps were performed at 0°C. The supernatant was then removed, and the mycelia were washed twice with 20 mL of buffer A (Tris-HCl (pH 7.5, 40 mM), 10% glycerol and 200 mM KCl and disrupted by sonication for 30 min. After centrifugation (12,000 × g; 25 min) at 4°C, the supernatant (cell-free extract) was collected for enzyme activity assays.

Approximately 400 μL of cell-free extract was incubated with kanamycin B (2 mM) in buffer A to a total volume of 500 μL at 28°C for 18 h. After all the enzymatic reactions, the protein was precipitated with chloroform (500 μL), and the precipitate was removed by centrifugation (7,160 × g; 10 min). The supernatant was analyzed by HPLC. Boiled cell-free extracts were used as negative controls, and these assay mixtures were subjected to the same experimental procedures and analyzed in parallel with those of the corresponding cell-free extract samples. The difference between the amount of kanamycin A produced in a sample and that in a control was caused by the enzyme reaction. The changes in KanJ and KanK activity can be roughly estimated based on the change in kanamycin A production. The amount of kanamycin A produced in the sample of the original strain minus that in the negative control was used as a reference against to the activities of all the other samples were related. (The amount in the original strain was arbitrarily assigned a value of 1, and the ratios of the other strains to the original strain are shown.) Experiments were performed with three independent samples, and error bars in the figures indicate the SD.

### Analysis of KanJ and KanK gene transcription

The original strain and *S*. *kanamyceticus* JKE1, JKE2, JKE3 and JKE4 were cultured in agar-free sporulation medium at 28°C for 96 h. The fermentation broth was then pooled (approximately 20 mL) in a 50-mL polypropylene tube. The mycelia were recovered by centrifugation (12,000 × g; 30 min) at 0°C, and RNA samples were obtained using Trizol Total RNA Extraction Kit (Probegene, China). To remove any genomic DNA contamination, the RNA preparations were digested with RNase-free DNase I (TaKaRa, Japan). The quality and quantity of the RNA samples were assessed by 1% agarose gel electrophoresis and spectrophotometry.

The PrimeScript RT Reagent Kit (TaKaRa, Japan) and SYBR Premix Ex Taq II Kit (TaKaRa, Japan) were used according to the instructions provided by the manufacturer. Primers JQ1 and JQ2, KQ1 and KQ2, listed in S1 Table, were used to generate PCR products. The real-time RT-PCR (qRT-PCR) reactions were carried out in the StepOnePlus real-time PCR system (Life Technologies, USA), using the following conditions: 95°C for 2 min, followed by 40 cycles of 95°C for 20 s, 60°C for 20 s, and 72°C for 20 s. The transcription levels of the tested genes were normalized to the internal control 16S rRNA and then determined using the 2^-ΔΔCT^ method [22]. The transcript level of each gene in the original strain was arbitrarily assigned a value of 1. The qRT-PCR experiments were performed with three independent RNA samples (biological replicates), and error bars indicate the SD.

## Results

### *kanN* was disrupted to delineate the kanamycin biosynthetic pathway in *S*. *kanamyceticus*

KanN is a 2'-*N*-acetylparomamine deacetylase, and according to the proposed parallel biosynthetic pathway, this enzyme is only involved in kanamycin B synthesis but not in kanamycin A synthesis. Based on this proposed pathway, *kanN* disruption will result in kanamycin B accumulation, whereas based on the linear pathway, 2'-*N*-acetylparomamine, the substrate of KanN, should accumulate.

An in-frame deletion strategy was used to inactivate *kanN* in order to eliminate any possible polar effects on other genes. The *kanN* in-frame deletion plasmid pDLA302 was introduced into *S*. *kanamyceticus* CG305 by conjugation transfer to obtain a *kanN*-disruption strain via the double-crossover homologous recombination event. The results confirmed that the selected strain named *S*. *kanamyceticus* Δ*kanN* does not possess the 705-bp *kanN* in-frame sequence (S1 Fig). The phenotypic characteristics of *S*. *kanamyceticus* Δ*kanN*, including colony color and shape, mycelium morphology, and growth rates, were not changed in comparison with the original strain.

**Fig 2:**
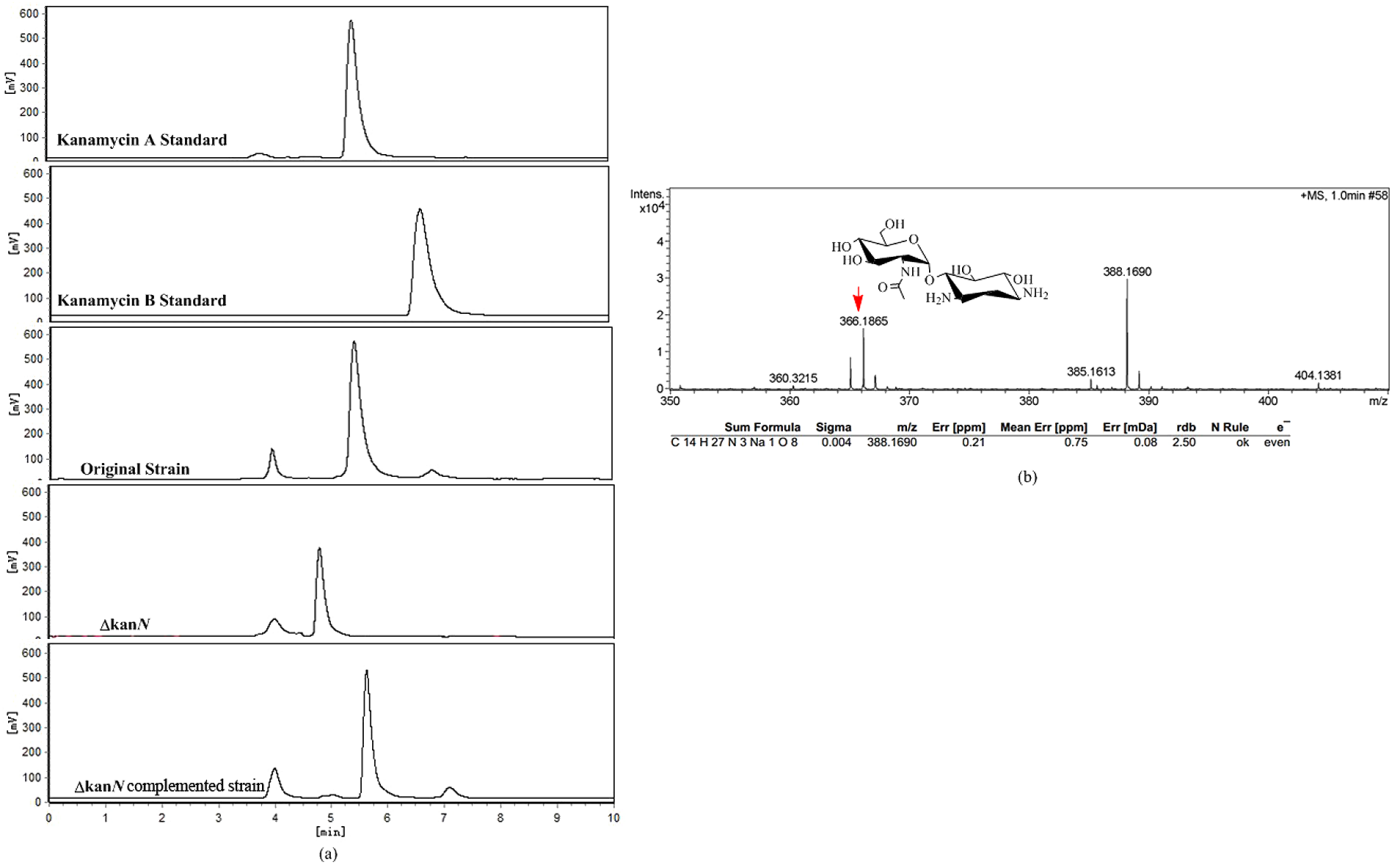
Metabolite analysis of *S*. *kanamyceticus* CG305 and *S*. *kanamyceticus* Δ*kanN*. **(a)** HPLC analysis of metabolites. **(b)** HPLC-MS analysis of the products of *S*. *kanamyceticus* Δ*kanN*.

Subsequently, *S*. *kanamyceticus* Δ*kanN* was fermented, and its products were analyzed. The original strain was used as a control under the same conditions. HPLC analysis demonstrated that kanamycin A and kanamycin B were included in the products of the original strain but were not found among those of *S*. *kanamyceticus* Δ*kanN*. The product of *S*. *kanamyceticus* Δ*kanN* included only one peak, with a retention time of 4.9 min (Fig 2A). High-resolution MS experiments demonstrated that this new compound has a molecular ion peak at 366 m/z and a chemical formula of C_18_H_37_N_4_O_11_, which is consistent with the metabolite 2'-*N*-acetylparomaine (Fig 2B).

To exclude the possibility of polar effects in the disruption mutant, the intact *kanN* gene in *S*. *kanamyceticus* Δ*kanN* was restored. We constructed the pLNE02 complementation plasmid containing *kanN*. When pLNE02 was introduced into the *kanN*-disruption strain, kanamycin A was synthesized, indicating that the production of 2'-*N*-acetylparomamine in *S*. *kanamyceticus* Δ*kanN* was due to the absence of *kanN* (Fig 2A).

The accumulation of 2'-*N*-acetylparomamine in the fermentation product of *S*. *kanamyceticus* Δ*kanN* suggested that kanamycin biosynthesis might not proceed through a parallel pathway *in vivo*.

### *kanJ* was disrupted in *S*. *kanamyceticus* to obtain a kanamycin B-overproducing strain

To demonstrate that KanJ and KanK convert kanamycin B to kanamycin A *in vivo*, *kanJ* was disrupted in the original strain. Thus, we can target *kanJ* and *kanK* to improve kanamycin-producing strains.

An in-frame deletion strategy was used to inactivate *kanJ* in order to eliminate any possible polar effects on other genes. The *kanJ* in-frame deletion plasmid pLDJ202 was introduced into *S*. *kanamyceticus* CG305 by conjugation transfer to obtain the *kanJ-*disruption strain via the double-crossover homologous recombination event. The results confirmed that the selected strain designated *S*. *kanamyceticus* Δ*kanJ* does not possess the 823-bp *kanJ* in-frame sequence (S3 Fig). The phenotypic characteristics of *S*. *kanamyceticus* Δ*kanJ*, including colony color and shape, mycelium morphology, and growth rates, were not altered in comparison with the original strain.

**Fig 3:**
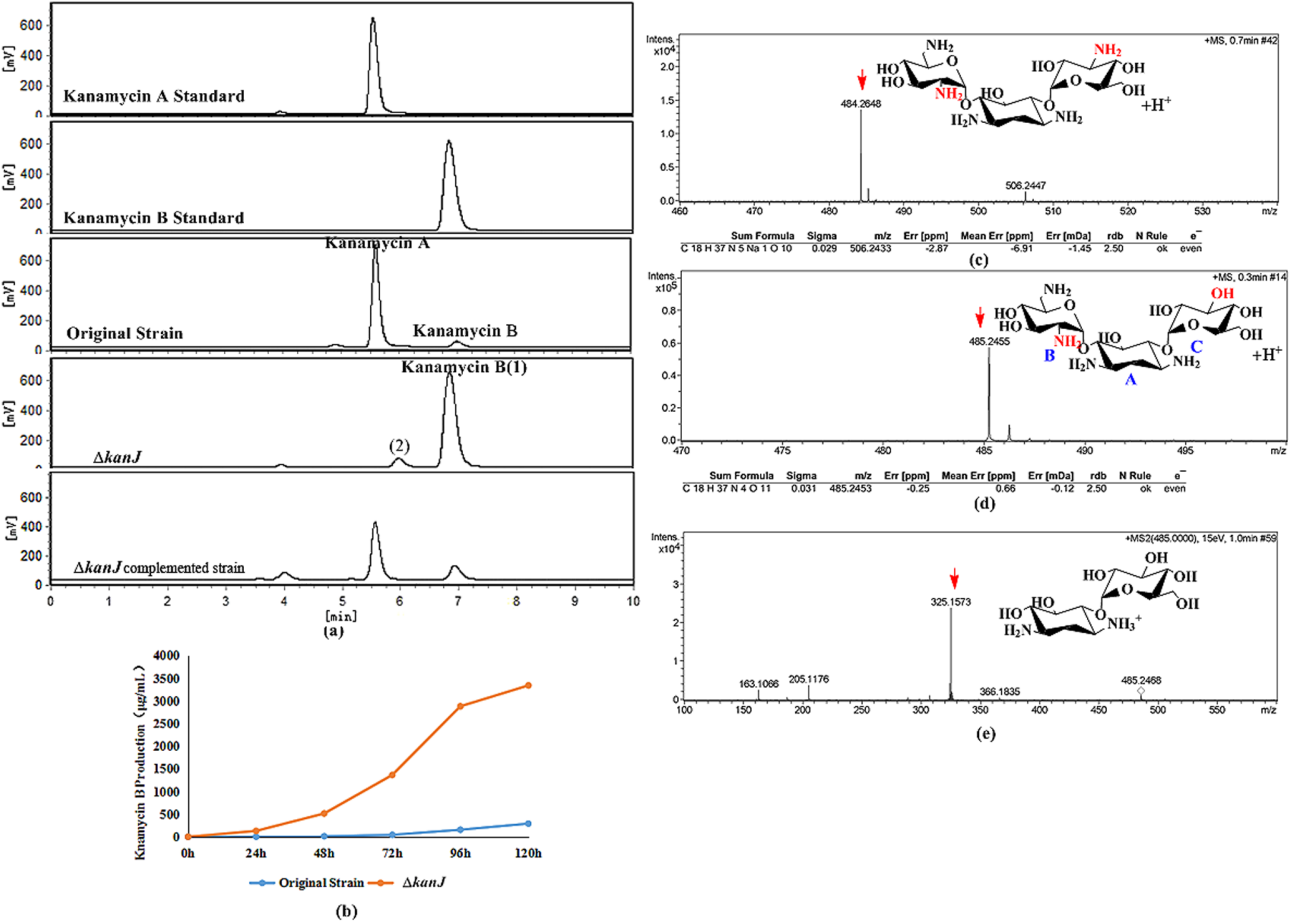
Metabolite analysis of *S*. *kanamyceticus* CG305 and *S*. *kanamyceticus* Δ*kanJ*. **(a)** HPLC analysis of metabolites. **(b)** Time-course of kanamycin B production. **(c)** HPLC-MS analysis of the main product (1) of *S*. *kanamyceticus* Δ*kanJ*. **(d)** HPLC-MS analysis of the byproduct (2) of *S*. *kanamyceticus* Δ*kanJ*. The blue letters indicate the number of carbon rings. **(e)** HPLC-MS/MS analysis of the byproduct (2) of *S*. *kanamyceticus* Δ*kanJ*.

Subsequently, the *S*. *kanamyceticus* Δ*kanJ* was fermented, and its products were analyzed. The original strain was used as the control under the same conditions. HPLC analysis demonstrated that the products did not contain kanamycin A, and the main component of these products had the same retention time as kanamycin B (Fig 3A). HPLC-MS demonstrated that the main component of the fermentation product had a molecular ion peak at 484.2648 m/z and a chemical formula of C_18_H_37_N_5_O_10_ (Fig 3C), which is consistent with kanamycin B. NMR analysis revealed that the chemical shifts of the main product were identical to those of kanamycin B (S2 Table & S9 Fig) [23]. This result confirmed that *S*. *kanamyceticus* Δ*kanJ* mainly produces kanamycin B.

The kanamycin B yield of *S*. *kanamyceticus* Δ*kanJ* was 3268±255 μg/mL (Fig 3B).

The fermentation of *S*. *kanamyceticus* Δ*kanJ* produced a byproduct, and high-resolution MS demonstrated that this byproduct had a molecular ion peak at 484.2655 m/z and a chemical formula of C_18_H_37_N_4_O_11_ (Fig 3D)_._ Three kanamycin secondary metabolites, namely kanamycin A, kanamycin C, and 3′′-deamine-3′-hydroxykanamycin B, have this formula. Because ring C (Fig 3D) has no amino groups, 3′′-deamine-3′′-hydroxykanamycin B generates a fragment peak at 325 m/z after glycosidic bond rupture, whereas kanamycin A and kanamycin C only show fragment peaks at 324 m/z. However, a fragment peak at 325 m/z was detected by MS/MS (Fig 3E), which confirmed that *S*. *kanamyceticus* Δ*kanJ* produced a minimal amount of 3′′-deamine-3′′-hydroxykanamycin B. In the downstream purification steps, the byproduct was isolated with a strong acid cation-exchange resin 001 × 7 (Anhui Sanxing Resin Technology), and the bound substance was eluted with 0.3 mol/L NH_4_OH. 74.3% of the kanamycin B was recovered at a purity of 94.5%, making it readily available for industrial production.

To exclude the possibility of polar effects in the disruption mutant, the intact *kanJ* gene was restored in the *S*. *kanamyceticus* Δ*kanJ* strain. We constructed the pLJE02 complementation plasmid containing *kanJ*. When pLJE02 was introduced into the *kanJ*-disruption strain, kanamycin A was synthesized, indicating that the difference in fermentation products of *S*. *kanamyceticus* Δ*kanJ* was due to the absence of *kanJ* (Fig 3A).

Based on the proposed parallel pathway, *S*. *kanamyceticus* Δ*kanJ* should be able to synthesize kanamycin A. However, *S*. *kanamyceticus* Δ*kanJ* mainly produces kanamycin B, without kanamycin A. Therefore, the *kanJ*-disruption experimental results confirmed that kanamycin biosynthesis does not proceed through the parallel pathway and that synthesis of kanamycin A from kanamycin B is catalyzed by KanJ in *S*. *kanamyceticus*.

The kanamycin B yield of *S*. *kanamyceticus* Δ*kanJ* reached 3268±255 μg/mL, which is 12-fold higher than that of the original strain (Fig 3B). This result proved that we successfully constructed the kanamycin B-high-yield strain *S*. *kanamyceticus* Δ*kanJ*.

### Overexpression of *kanJ* and *kanK* in *S*. *kanamyceticus* minimized kanamycin B as a byproduct of kanamycin A production

Because the synthesis of kanamycin A *in vivo* is due to *kanJ* and *kanK*, increased expression of *kanJ* and *kanK* can promote kanamycin B conversion to kanamycin A. To obtain an optimal kanamycin A-producing strain with a lower kanamycin B yield, *kanJ* and *kanK* were therefore overexpressed in *S*. *kanamyceticus* CG305.

**Fig 4:**
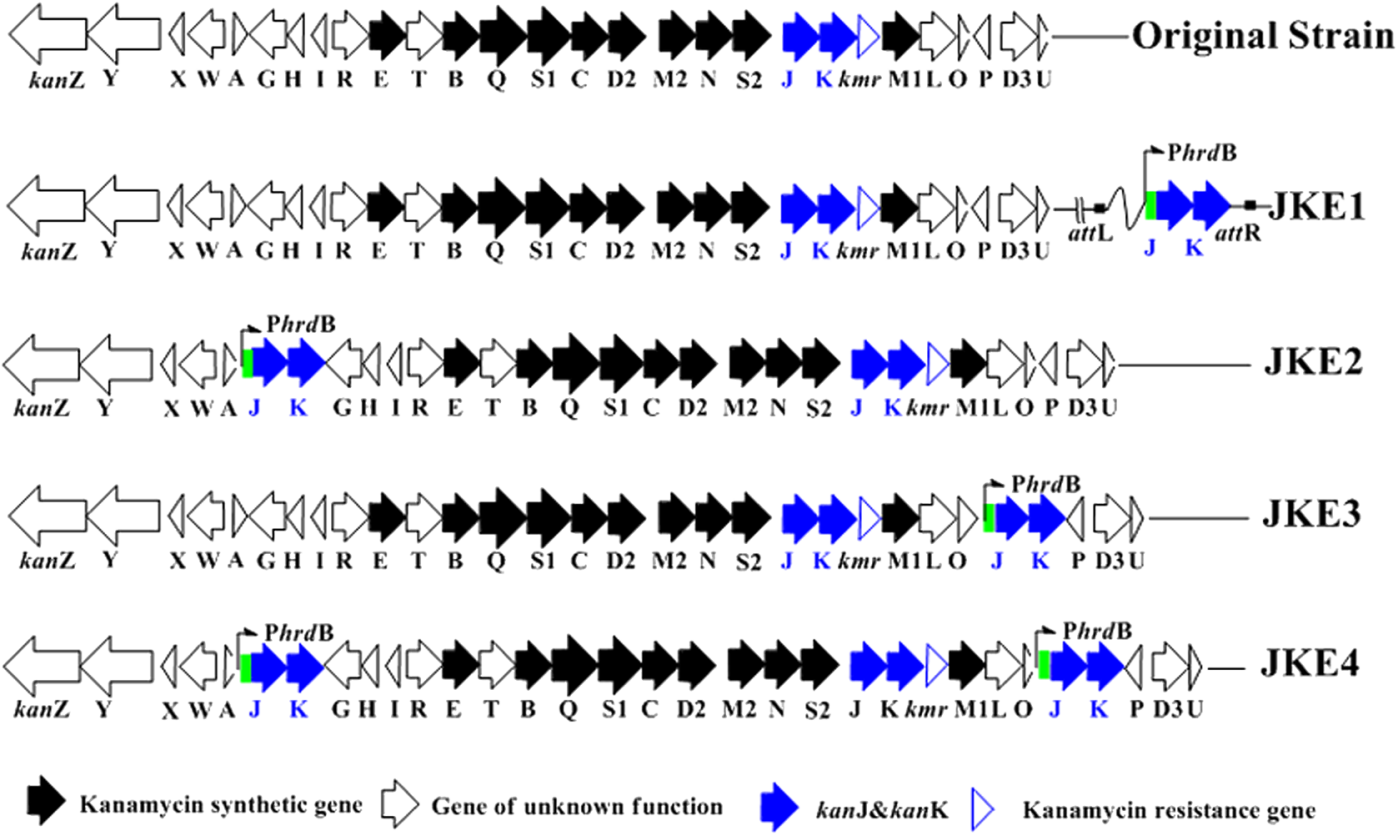
Genotypes of *Streptomyces kanamyceticus* CG305 and its recombinant overexpressing strains.

For the efficient expression of *kanJ* and *kanK*, *PhrdB*, which has been confirmed to be stronger than PermE* in several other *Streptomyces* strains, was used as the promoter [24]. The overexpression plasmid pHJK1, which includes *PhrdB*, *kanJ* and *kanK* was constructed and introduced into *S*. *kanamyceticus* CG305. The overexpression strain was denoted *S*. *kanamyceticus* JKE1, and its genotype was confirmed by PCR analysis (Fig 4 and S5 Fig). Concurrently, pEAP1 without a homologous gene was introduced into *S*. *kanamyceticus* CG305 to obtain the control strain. The content and composition of the metabolites of the control strain showed no significant differences compared with the metabolites of the original strain (data not shown).

**Fig 5:**
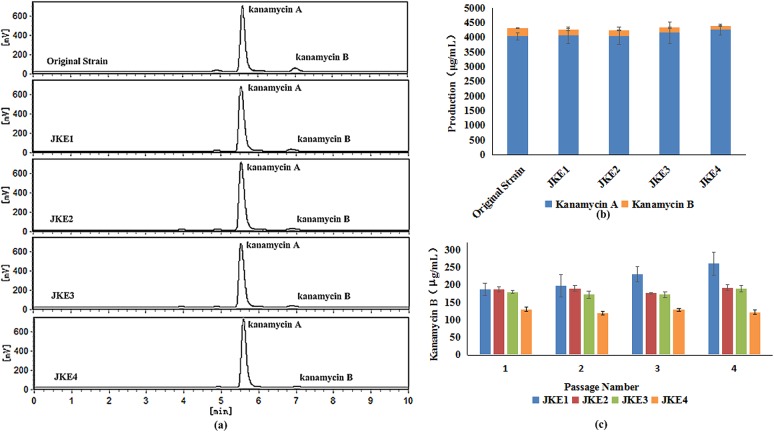
Metabolites of *S*. *kanamyceticus* CG305 and its recombinant overexpressing strains. **(a)** HPLC analysis of metabolites. **(b)** Analysis of secondary metabolite yields. **(c)** Stability of kanamycin B yields from *S*. *kanamyceticus* JKE1, JKE2, JKE3, and JKE4.

Subsequently, *S*. *kanamyceticus* JKE1 was fermented, and its products were analyzed. HPLC assays demonstrated that the kanamycin B yield decreased from 278±13 μg/mL to 187±30 μg/mL and that the percentage of kanamycin B among the total kanamycin products decreased from 6.42% to 4.43%. The kanamycin A yield of the original strain was 4039±122 μg/mL and that of *S*. *kanamyceticus* JKE1 was 4084±284 μg/mL (Fig 5A and 5B).

The genetic stability of strains used for industrial fermentation is extremely important. However, over four generations of unselected passages, the kanamycin B production of *S*. *kanamyceticus* JKE1 increased from 187±30 μg/mL to 260±33 μg/mL (Fig 5C). To determine the stability of the overexpression plasmid, 36 single colonies were randomly selected from each generation, and their genomic DNA was examined by PCR. The results revealed that more than 95.3% (±3%) of the colonies eliminated the overexpression plasmid from the chromosome after four generations of unselected passage (S5 Fig). Simultaneously, the control strain, which contained only pEAP1, also showed elimination of the plasmid. Thus, we speculate that the change in yield might be due to elimination of the plasmid and that this is a systematic issue.

To avoid genetic instability, we integrated *kanJ* and *kanK* into the chromosome via homologous recombination rather than site-specific integration. To ensure that *kanJ* and *kanK* were inserted into an active area of the chromosome without inducing polar effects, we inserted these gene between the two reverse genes in the biosynthetic gene cluster. The first pair of genes selected was *kanA* and *kanG*. Then, *kanJ* and *kanK*, which are under the control of *PhrdB*, were inserted into the *S*. *kanamyceticus* CG305 chromosome via homologous recombination. The overexpressing strain was designated *S*. *kanamyceticus* JKE2 (Fig 4). Simultaneously, the *kanJ* and *kanK* genes were inserted between *kanO* and *kanP* in the *S*. *kanamyceticus* CG305 chromosome using the same method, and the resulting strain was designated *S*. *kanamyceticus* JKE3 (Fig 4). The kanamycin B yields of *S*. *kanamyceticus* JKE2 and *S*. *kanamyceticus* JKE3, as determined by HPLC, were 187±13 and 181±22 μg/mL, corresponding to 4.40 and 4.17% of the total kanamycin yield of each strain, respectively, and these values are lower than those of the original strain. Kanamycin A yields were 4059±204 and 4161±269 μg/mL, respectively (Fig 5B). As a result, the yield of kanamycin B decreased, and the purity of kanamycin A in the fermentation product improved. The production of kanamycin B by *S*. *kanamyceticus* JKE2 and *S*. *kanamyceticus* JKE3 was stable over four generations of unselected passages (Fig 5C).

To further reduce the yield of kanamycin B, the *kanJ* and *kanK* genes were inserted between *kanO* and *kanP* in the *S*. *kanamyceticus* JKE2 strain. Therefore, we constructed *S*. *kanamyceticus* JKE4, an overexpression strain containing three copies of the target genes. An HPLC assay indicated that the kanamycin B yield was further reduced to 128±20 μg/mL and that the kanamycin A yield was 4266±191 μg/mL. The percentage of kanamycin B in the products (2.94%) was also reduced compare with that of the original strain (4.4%). The kanamycin B yield was further decreased, and the purity of kanamycin A in the fermentation products was improved. Moreover, the stability of kanamycin B production by *S*. *kanamyceticus* JKE4 was confirmed (Fig 5C).

**Fig 6:**
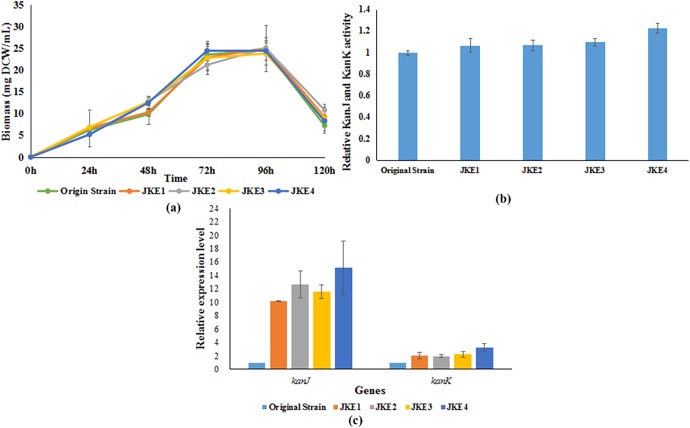
Cell growth, relative enzyme activity and qRT-PCR gene transcription analysis of kanamycin A-producing strains. **(a)** Cell growth of kanamycin A-producing strains. **(b)** The relative activity of KanJ and KanK in mycelia after fermentation for 72 h. **(c)** qRT-PCR gene transcription analysis of *kanJ* and *kanK* in the original strain and in the *S*. *kanamyceticus* JKE1, JKE2, JKE3 and JKE4 strains.

As shown in Fig 6A, the growth curves of *S*. *kanamyceticus* JKE1, JKE2, JKE3 and JKE4 were almost identical to those of the original strain. In particular, the genetic manipulations performed in this study had no effect on the mycelial growth of the strains. The pre-experimental results showed that KanJ and KanK were most active after 72 h of culture. Thus, after 72 h of fermentation, the broths were sonicated to determine the relative activity of KanJ and KanK. The samples were then analyzed by HPLC. The sample of *S*. *kanamyceticus* Δ*kanJ* had no kanamycin A, indicating that all of the kanamycin A was converted by KanJ and KanK. Thus, the changes in KanJ and KanK activity can be roughly estimated based on the change in the kanamycin A level. The relative activities of KanJ and KanK increased in the samples of the *S*. *kanamyceticus* overexpression strains JKE1, JKE2, JKE3, and JKE4 compared with that in the original strain. The relative activities of KanJ and KanK in the *S*. *kanamyceticus* JKE4 sample were higher than those of the other samples (Fig 6B). The KanJ and KanK activity of this strain equaled 120% that of the activity of the original strain, and this increase was likely due to the higher copy number and greater expression of *kanJ* and *kanK* in *S*. *kanamyceticus* JKE4. Thus, the kanamycin B yield of *S*. *kanamyceticus* JKE4 was relatively lower than that of the other strains.

The expression levels of *kanJ* and *kanK* were assessed via qRT-PCR. As shown in Fig 6C, the qRT-PCR results indicated that the expression levels of *kanJ* and *kanK* in the original strain were lower than those in the other strains under the same culture conditions. The expression level increase of *kanJ* was higher than that of *kanK* in the overexpressing strains (Fig 6C), probably due to the fact that the *kanJ* gene fragment is closer to *PhrdB* promoter. In addition, the highest-expression levels were detected in *S*. *kanamyceticus* JKE4, consistent with the results of the fermentation and enzyme activity assays.

An optimal kanamycin A-producing strain, which produces a higher purity of kanamycin A and a lower kanamycin B yield, was thus constructed. As a result, the required separation and extraction processes can be significantly simplified, and production costs can be reduced.

## Discussion

Based on biochemical experiments [16] and studies of heterologous expression in *S*. *venezuelae* [6], researchers have proposed two different biosynthetic pathways for kanamycin A. Based on the parallel biosynthetic pathway, 2'-*N*-acetylparomamine deacetylase, known as KanN, is involved in the biosynthesis of kanamycin B but not the biosynthesis of kanamycin A. As a result, *kanN* disruption should generate a strain that produces high yields of kanamycin A. However, the accumulation of only 2'-*N*-acetylparomamine (and no kanamycin A accumulation) in the fermentation products of *S*. *kanamyceticus* Δ*kanN* suggests that *in vivo* kanamycin biosynthesis might not follow the parallel pathway. Moreover, based on the proposed parallel biosynthetic pathway, the *S*. *kanamyceticus* Δ*kanJ* strain, in which the C-2' dioxygenase *kanJ* gene is disrupted, should synthesize kanamycin A. An analysis of the fermentation products of this strain revealed that *S*. *kanamyceticus* Δ*kanJ* is unable to produce kanamycin A components and generates only kanamycin B components. Therefore, our experimental results clarified that kanamycin biosynthesis does not proceed through the parallel pathway and that kanamycin A is obtained through the conversion of kanamycin B by KanJ and KanK (linear pathway) in *S*. *kanamyceticus*. Increased expression of *kanJ* and *kanK* can promote the conversion of kanamycin B to kanamycin A. Similarly, *kanJ* and *kanK* were expressed in *M*. *echinospora* for gentamicin B production, and the results showed the C-2' hydroxy synthesis activity of KanJ and KanK [25].

The byproduct 3′′-deamine-3′′-hydroxykanamycin B was detected in the fermentation products of the *kanJ*-disruption strain. Kanamycin B has an amino group at position C3′′, whereas the by-product 3′′-deamine-3′′-hydroxykanamycin B possesses a hydroxyl group at the same position. The difference is the 3-amino group on the third sugar moiety. In 2011, Park et al [6] found that UDP-Glc can be converted to UDP-Kns by KanD2 and KanS2. UDP-Kns becomes the glycosyl donor in the trisaccharide synthesis reaction catalyzed by KanM2. The results of heterologous expression showed that, although the optimal substrate for KanM2 is UDP-Kns, KanM2 also has the ability to utilize UDP-Glc [6]. The accumulation of kanamycin B may alter the selectivity of KanM2, leading to the selection of a small amount of UDP-Glc as a substrate for the generation of 3′′-deaminated-3′′-hydroxy kanamycin B. In the original strain, kanamycin A is continuously synthesized from kanamycin B, resulting in no detectable byproduct accumulation.

In the second part of this study, the yields of *S*. *kanamyceticus* JKE2, JKE3 and JKE4 were found to be more stable than that of *S*. *kanamyceticus* JKE1. Researchers attempting to manipulate the genes of the strain that produce gentamicin, an aminoglycoside antibiotic, encountered the same phenomenon [25]. In our experiment, as both *S*. *kanamyceticus* JKE1 and the control strain showed the elimination of plasmid, we speculate that the change in yield might be caused by elimination of the plasmid and that this is a systematic issue. Though we tried our best to pick the integrated strain (The expected genotype was first identified by erythromycin resistance and confirmed by PCR using primers located outside of the exchange regions (S5 Fig). Extraction of free plasmids was then performed, and no free plasmid was obtained.), the possibility of free plasmids presence cannot be completely ruled out. Therefore, it is possible that the elimination of plasmid is due to free plasmids. Moreover, the plasmid instability issue can occur when a φC31-derived plasmid is used in a strain containing multiple pseudo *attB* sites [26]. In addition, the C-terminus of the φC31 integrase contains the DNA-binding domain (aa 300–500), the conformation of which controls the directionality of integration versus excision [27]. Recently, we sequenced the integrase gene of PHJK1 and found that aa 310, a Thr, is mutated to an Asn, which may also be a cause of plasmid instability. The qRT-PCR results shown that the expression level increase of *kanJ* was higher than that of *kanK* in the overexpressing strains, probably due to the fact that the *kanJ* gene fragment is closer to *PhrdB* promoter. In subsequent experiments, by further enhancing the expression of *kanK*, it should be possible to further promote kanamycin B conversion to kanamycin A. Moreover, expression of *kanJ* and *kanK* and the production of kanamycin B by the three overexpressing strains containing two copies of *kanJ* and *kanK* (*S*. *kanamyceticus* JKE1, JKE2 and JKE3) were similar, whereas the genomic positions of the target fragment were different in the various strains. This result indicates that compared to relative position with *PhrdB* promoter, the genomic position of *kanJ* and *kanK* is not a key factor in determining expression efficiency in *S*. *kanamyceticus*. The gene can be effectively expressed regardless of whether it is integrated within different regions of the gene cluster or outside the gene cluster.

Many experiments have confirmed that increasing the copy number of a particular biosynthetic gene can increase the yield of and eliminate impurities from the desired product. Chen et al. [28] altered the gene copy numbers of *eryK* and *eryG* in *Saccharopolyspora erythraea*. Specifically, at an *eryK* and *eryG* copy number ratio of 3:2, erythromycin B and erythromycin C were nearly eliminated and subsequently converted to erythromycin A. In another study, to improve L-tryptophan production, Gu et al. [29] expressed *aroK*, which plays a pivotal role in aromatic amino acid biosynthesis. The yields of the strains that contained different copy numbers of *aroK* were examined, and the results indicated that the presence of two integrated copies (three copies in total) of *aroK* resulted in the highest yields. A greater number of gene copies might increase the metabolic burden of a cell, which is not conducive to the production of the desired products. In our experiments, *kanJ* and *kanK* were overexpressed in *S*. *kanamyceticus*, and in the overexpressing strain containing three copies of *kanJ* and *kanK*, the purity of kanamycin A increased, and the yield of kanamycin B decreased by 54%. These results demonstrated that the overexpression of essential enzymes enhances the biotransformation process and selectively improves the production and purity of the desired components in the fermentation stage.

## Conclusion

To obtain a kanamycin B-high-yield strain and an optimal kanamycin A-producing strain, we delineated the kanamycin A biosynthetic pathway in *S*. *kanamyceticus* in this study. The *kanN*-disruption mutant *S*. *kanamyceticus* Δ*kanN* only produced 2'-*N*-acetylparomamine, whereas the *kanJ-*disruption strain *S*. *kanamyceticus* Δ*kanJ* produced kanamycin B as its main metabolite. These results clarified that kanamycin A is derived from the conversion of kanamycin B catalyzed by KanJ and KanK (linear pathway) in *S*. *kanamyceticus*. As expected, the kanamycin B yield of *S*. *kanamyceticus* Δ*kanJ* increased from 278±13 μg/mL to 3268±255 μg/mL, which was 12-fold higher than that of the original strain. By overexpressing the *kanJ* and *kanK* genes, we ultimately obtained an optimal kanamycin A-producing strain showing minimal kanamycin B byproduct accumulation, with a 54% lower kanamycin B yield.

## Supporting information

S1 Table Primers used in this study. (DOCX)

S2 Table ^13^C-NMR (100 MHz) chemical shifts for kanamycin B [24] and the main products of *S*. *kanamyceticus* Δ*kanJ* in D_2_O. (DOCX)

S1 Fig Disruption experiment of *kanN*. (DOCX)

S2 Fig Complementation experiment of *S*. *kanamyceticus* Δ*kanN*. (DOCX)

S3 Fig Disruption experiment of *kanJ*. (DOCX)

S4 Fig Complementation experiment of *S*. *kanamyceticus* Δ*kanJ*. (DOCX)

S5 Fig Construction of the *kanJ-* and *kanK-*overexpressing strain *S*. *kanamyceticus* JKE1. (DOCX)

S6 Fig Construction of the *kanJ-* and *kanK-*overexpressing strain *S*. *kanamyceticus* JKE2. (DOCX)

S7 Fig Construction of the *kanJ-* and *kanK-*overexpressing strain *S*. *kanamyceticus* JKE3. (DOCX)

S8 Fig Construction of the *kanJ-* and *kanK-*overexpressing strain *S*. *kanamyceticus* JKE4. (DOCX)

S9 Fig ^13^C NMR spectrum of the main products of *S*. *kanamyceticus* Δ*kanJ*. (DOCX)
