# Supplementary material for: Modulation of kanamycin B and kanamycin A biosynthesis in Streptomyces kanamyceticus via metabolic engineering
Source: PLoS One. 2017 Jul 28;12(7):e0181971. doi: 10.1371/journal.pone.0181971 (PMC5533434; doi:10.1371/journal.pone.0181971)
Supplement: S1 Table — (DOCX) [file pone.0181971.s001.docx]

**S1 Table.** **Primers used in this study^a^**

| Name | Sequence |
| --- | --- |
| AP1III | 5′- CCAAGCTTTCGACCTGTGGACCGGCATGGGGCT-3′ |
| AP2III | 5′-CGGAATTCTCGGGGTGGGGGGAGACGAGGAGGA-3′ |
| AP3III | 5′- CGGAATTCGCCGCCGTGACACCCGACAA -3′ |
| AP4III | 5′-CGGGATCCGCCCGGCAGGTTCATGTTGC-3′ |
| AY1 | 5′- CGGGCAAGCACGTACTGGTGGAGA-3′ |
| AY2 | 5′- ACAGCGCCAGCTTCTTGCTCATAG-3′ |
| AY3 | 5′- TCGACCGAGAGGCAGTACGGGGGGT-3′ |
| AY4 | 5′- GAATCGTCGTACCCCCGTTCAGGA-3′ |
| AE1 | 5′-GGATCCAGCA GTGAGGGTCCTCCTCGTCTCCCCCCA-3′ |
| AE2 | 5′-AAGCTTCGCCAGCTTCTTGCTCATAGCGACTCC-3′ |
| E1 | 5′-TATTCTAGAGGTACCAGCCCGACCCGAGCACGCG-3′ |
| JII1 | 5′- CCAAGCTTAGGAGTCGCTATGAGCAAGAAG-3′ |
| JII2 | 5′- CGGAATTCATGGCTGGTCTTCCCTTCTCA-3′ |
| JII3 | 5′- CGGAATTCCCTGACGGGTGATCATCCCTT-3′ |
| JII4 | 5′-CCAAGCTTGCGGAATGGGTCTGGTACATG-3′ |
| JY1 | 5′-CGTCTTCGGCACGACCCTGTAC-3′ |
| JY2 | 5′-TTGCAAAGATTGGCCGATAACTC-3′ |
| JY3 | 5′-TACTACGAGGTCATCTACCGCTTC-3′ |
| JY4 | 5′-GACATCCGCAGGATCTGTACCTG-3′ |
| JE1 | 5′-CGGGATCCAGCAATGGCCCTTGCCGCTCCG-3′ |
| JE2 | 5′-CCAAGCTTGCCCCCGCAGAGCGAGTTGACTG-3′ |
| H1 | 5'-GAATTCCGCCTTCCGCCGGAACG-3' |
| H2 | 5'-CCATGGACAACCTCTCGGAACGTTG-3' |
| JKE1 | 5′-CCATGGCCCTTGCCGCTCCGCC-3′ |
| JKE2 | 5′- CCAAGCTTGAATTCGTGAGCCCGCCCCTCCCGTGACGA-3′ |
| PR1 | 5′- CGGAATTCGGACAGATGGGCACTCGCATAGACG-3′ |
| PR2 | 5′- CGGGATCCCTGGGACAGCGTGAACATCTCTGGT-3′ |
| PR3 | 5′- CGGAATTCTCTAGAGCTACAGCAAGGTGGGCTTCAAG-3′ |
| PR4 | 5′- CCAAGCTTGACCCGTGGGGGCTGACTTG-3′ |
| PR5 | 5′- GGGGTACCGAGGCAGGACTGGCAGCACTTGGA-3′ |
| PR6 | 5′- CGGGATCCCGCACGACCCGTTCGGCAAGA-3′ |
| PR7 | 5′- CGGGATCCTGGACGAGGACGGCAAGCTGGT-3′ |
| PR8 | 5′- CCAAGCTTCCTGGTTGAGGTAGCCGAAGAGCA-3′ |
| R13JK1 | 5′- GGAGAGCCAACCACCCTGCGTA-3′ |
| R13JK2 | 5′- CCGAGTCACACCCCGTAAGCGA-3′ |
| R13JK3 | 5′- ACCTGAGGAGGACACTTTGCGG-3′ |
| R13JK4 | 5′- CTACTACCGATGCCCCGCTCAA-3′ |
| R57JK1 | 5′- CGGGTTCCTGCCCAACGACGAGAT-3′ |
| R57JK2 | 5′- GGGTCTTGTCGTCCGGTGTGAGGG-3′ |
| R57JK3 | 5′- CTTCCGAAGATCAGTTACGTTCCTC-3′ |
| R57JK4 | 5′- CTGGCGTTGCGTGAGGACTTGG-3′ |
| JQ1 | 5′- AGCCACCCCCCGTACTGCCT-3′ |
| JQ2 | 5′- CCCGCTCGTCGTAGCACTCG-3′ |
| KQ1 | 5′- GAGGTCGCCCTGTGGCTGAT-3′ |
| KQ2 | 5′- CGGGCAACTCCTGTATCTCC-3′ |

^a^Restriction enzyme site are indicated by single underlines
