# Supplementary material for: Modulation of kanamycin B and kanamycin A biosynthesis in Streptomyces kanamyceticus via metabolic engineering
Source: PLoS One. 2017 Jul 28;12(7):e0181971. doi: 10.1371/journal.pone.0181971 (PMC5533434; doi:10.1371/journal.pone.0181971)
Supplement: S2 Table — (DOCX) [file pone.0181971.s002.docx]

| Position | Kanamycin B | [Main](C:/Users/Administrator/AppData/Local/Yodao/DeskDict/frame/20160219111515/javascript:void(0);) [products](C:/Users/Administrator/AppData/Local/Yodao/DeskDict/frame/20160219111515/javascript:void(0);) of *S. kanamyceticus* Δ*kanJ* | Position | Kanamycin B | [Main](C:/Users/Administrator/AppData/Local/Yodao/DeskDict/frame/20160219111515/javascript:void(0);) [products](C:/Users/Administrator/AppData/Local/Yodao/DeskDict/frame/20160219111515/javascript:void(0);) of *S. kanamyceticus* Δ*kanJ* |
| --- | --- | --- | --- | --- | --- |
| 1 | 50.58 | 50.30 | 4′ | 71.55 | 71.26 |
| 2 | 35.43 | 35.23 | 5′ | 72.36 | 72.57 |
| 3 | 49.44 | 49.18 | 6′ | 41.57 | 41.40 |
| 4 | 86.10 | 86.22 | 1′′ | 100.14 | 100.04 |
| 5 | 74.60 | 74.30 | 2′′ | 71.90 | 71.66 |
| 6 | 88.30 | 87.79 | 3′′ | 54.39 | 54.08 |
| 1′ | 100.14 | 99.86 | 4′′ | 69.33 | 69.09 |
| 2′ | 55.41 | 55.18 | 5′′ | 72.21 | 71.90 |
| 3′ | 73.61 | 73.41 | 6′′ | 60.41 | 60.12 |

**S2 Table. ^13^C-NMR(100 MHz)chemical shifts for kanamycin B [24] and the** [**main**](C:/Users/Administrator/AppData/Local/Yodao/DeskDict/frame/20160219111515/javascript:void(0);) [**products**](C:/Users/Administrator/AppData/Local/Yodao/DeskDict/frame/20160219111515/javascript:void(0);) **of *S. kanamyceticus* Δ*kanJ* in D_2_O.**
