# Supplementary material for: Modulation of kanamycin B and kanamycin A biosynthesis in Streptomyces kanamyceticus via metabolic engineering
Source: PLoS One. 2017 Jul 28;12(7):e0181971. doi: 10.1371/journal.pone.0181971 (PMC5533434; doi:10.1371/journal.pone.0181971)
Supplement: S1 Fig — (DOCX) [file pone.0181971.s003.docx]

**S1 Fig. Disruption experiment of *kanN*.**


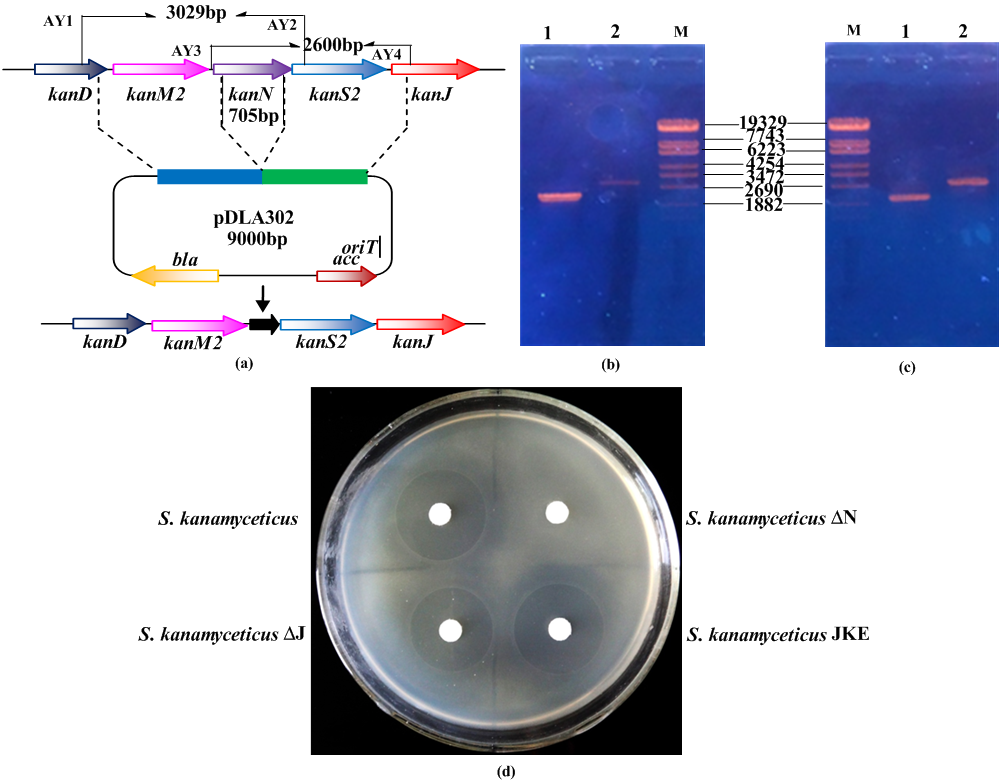


**N1 N2**

**(a)** Genotype of original strain *S. kanamyceticus* CG305 and mutant strain *S. kanamyceticus* Δ*kanN*. **(b)** PCR analysis with the genomic DNA from original strain and *S. kanamyceticus* Δ*kanN*, using primers AY1 and AY2 (indicated in (a)); 3029bp corresponding to intact *kanN* gene in original strain (lane 2) and 2324bp band (caused by deletion of 705 bp internal fragment in *kanJ*) in mutant strains *S. kanamyceticus* Δ*kanJ* (lane 1). Lane M indicates the DNA molecular weight marker (λ-*Eco*T14I digest). **(c)** PCR analysis with the genomic DNA from original strain and *S. kanamyceticus* Δ*kanN*, using primers AY3 and AY4 (indicated in (a)); 2600bp corresponding to intact *kanN* gene in original strain (lane 2) and 1895bp band (caused by deletion of 705 bp internal fragment in *kanN*) in mutant strain *S. kanamyceticus* Δ*kanN* (lane 1). Lane M indicates the DNA molecular weight marker (λ-*Eco*T14I digest).
