# Supplementary material for: Modulation of kanamycin B and kanamycin A biosynthesis in Streptomyces kanamyceticus via metabolic engineering
Source: PLoS One. 2017 Jul 28;12(7):e0181971. doi: 10.1371/journal.pone.0181971 (PMC5533434; doi:10.1371/journal.pone.0181971)
Supplement: S3 Fig — (DOCX) [file pone.0181971.s005.docx]

**S3** **Fig.** **Disruption experiment of *kanJ*.**


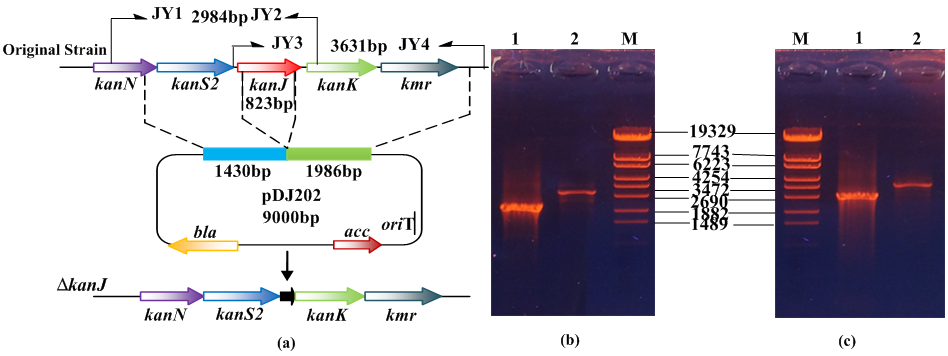


**J1 J2**

**(a)** Genotype of original strain *S. kanamyceticus* CG305 and mutant strain *S. kanamyceticus* Δ*kanJ*. **(b)** PCR analysis with the genomic DNA from original strain and *S. kanamyceticus* Δ*kanJ*, using primers JY1 and JY2 (indicated in (a)); 2984bp corresponding to intact *kanJ* gene in original strain (lane 2) and 2161bp band (caused by deletion of 823 bp internal fragment in *kanJ*) in mutant strains *S. kanamyceticus* Δ*kanJ* (lane 1). Lane M indicates the DNA molecular weight marker (λ-*Eco*T14I digest). **(c)** PCR analysis with the genomic DNA from original strain and *S. kanamyceticus* Δ*kanJ*, using primers JY3 and JY4 (indicated in (a)); 3631bp corresponding to intact *kanJ* gene in original strain (lane 2) and 2808bp band (caused by deletion of 823 bp internal fragment in *kanJ*) in mutant strain *S. kanamyceticus* Δ*kanJ* (lane 1). Lane M indicates the DNA molecular weight marker (λ-*Eco*T14I digest).
