# Supplementary material for: Modulation of kanamycin B and kanamycin A biosynthesis in Streptomyces kanamyceticus via metabolic engineering
Source: PLoS One. 2017 Jul 28;12(7):e0181971. doi: 10.1371/journal.pone.0181971 (PMC5533434; doi:10.1371/journal.pone.0181971)
Supplement: S4 Fig — (DOCX) [file pone.0181971.s006.docx]

**S4** **Fig. Complementation experiment of *S. kanamyceticus* Δ*kanJ*.**


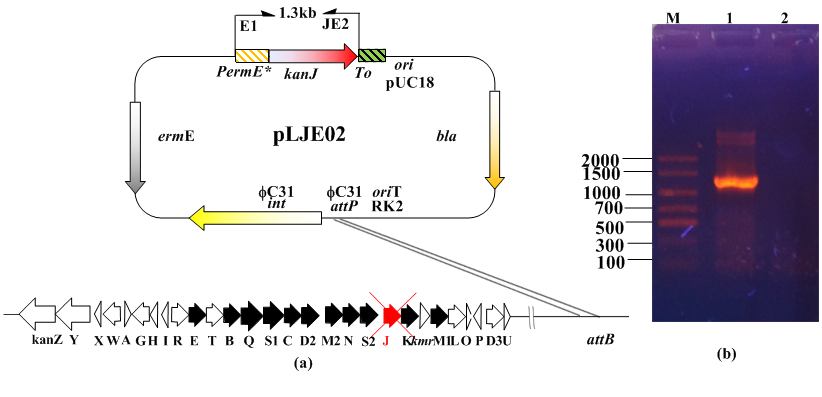


**(a)** Genotype of *S. kanamyceticus* Δ*kanJ* and map of genetic complementation vector **(b)** PCR analysis with the genomic DNA from *S. kanamyceticus* Δ*kanJ* and the *kanJ* genetically complemented strain, using primers E1 and JE2 (indicated in (a)); lane 1 is the *kanJ* genetically complemented strain and lane 2 is *S. kanamyceticus* Δ*kanJ*. Lane M indicates the DNA molecular weight marker (PCR DNA Ladder).
