# Supplementary material for: Modulation of kanamycin B and kanamycin A biosynthesis in Streptomyces kanamyceticus via metabolic engineering
Source: PLoS One. 2017 Jul 28;12(7):e0181971. doi: 10.1371/journal.pone.0181971 (PMC5533434; doi:10.1371/journal.pone.0181971)
Supplement: S5 Fig — (DOCX) [file pone.0181971.s007.docx]

**S5** **Fig. Construction of the *kanJ-* and *kanK-*overexpressing strain *S. kanamyceticus* JKE1.**


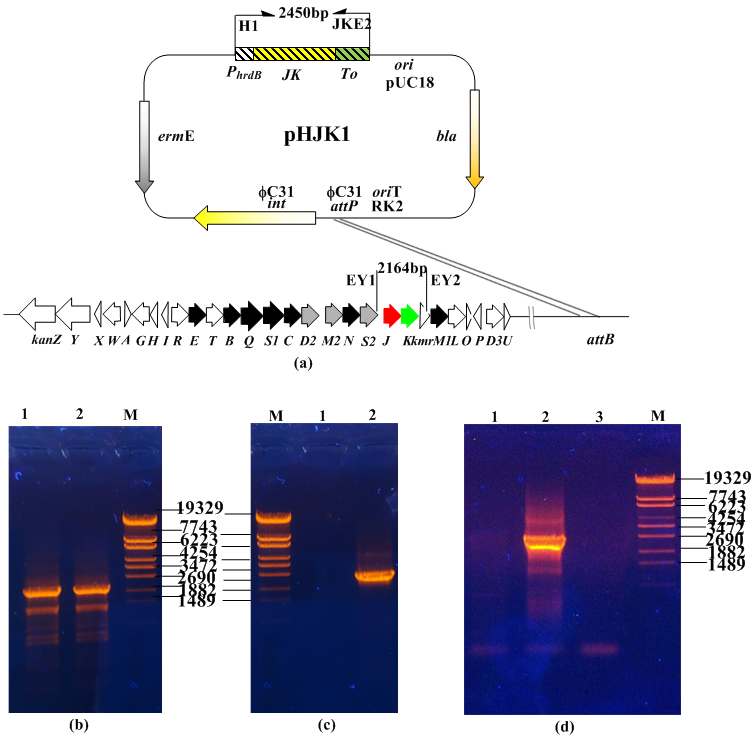


**(a)** Genotype of original strain *S. kanamyceticus* CG305 and mutant strain *S. kanamyceticus* JKE1. **(b)** PCR analysis with the genomic DNA from original strain and *S. kanamyceticus* JKE1, using primers EY1 and EY2 (indicated in (a)); lane 1 is original strain and lane 2 is *S. kanamyceticus* JKE1. Lane M indicates the DNA molecular weight marker (λ-*Eco*T14I digest). **(c)** PCR analysis with the genomic DNA from original strain and *S. kanamyceticus* JKE1, using primers H1 and JKE2 (indicated in (a)); lane 1 is original strain and lane 2 is *S. kanamyceticus* JKE1. Lane M indicates the DNA molecular weight marker (λ-*Eco*T14I digest). **(d)** PCR analysis with the genomic DNA from original strain, *S. kanamyceticus* JKE1 and the chromosome of *S. kanamyceticus* JKE1 after four generations of unselected passage, using primers H1 and JKE2 (indicated in (a)); lane 1, 2 and 3 is original strain, *S. kanamyceticus* JKE1 and selected single strain after four generations of unselected passage. Lane M indicates the DNA molecular weight marker (λ-*Eco*T14I digest)
