# Supplementary material for: Modulation of kanamycin B and kanamycin A biosynthesis in Streptomyces kanamyceticus via metabolic engineering
Source: PLoS One. 2017 Jul 28;12(7):e0181971. doi: 10.1371/journal.pone.0181971 (PMC5533434; doi:10.1371/journal.pone.0181971)
Supplement: S6 Fig — (DOCX) [file pone.0181971.s008.docx]

**S6** **Fig. Construction of the *kanJ-* and *kanK-*overexpressing strain *S. kanamyceticus* JKE2**

**
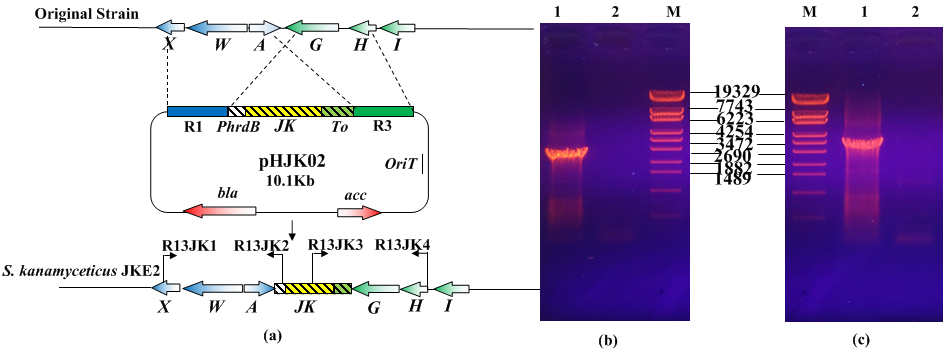
**

**(a)** Genotype of original strain *Streptomyces kanamyceticus* CG305 and mutant strain *S. kanamyceticus* JKE2. **(b)** PCR analysis with the genomic DNA from original strain and *S. kanamyceticus* JKE2, using primers R13JK1 and R13JK2 (indicated in (a)); 2584bp band corresponding to intact R1 and *PhrdB* promoter in *S. kanamyceticus* JKE2 (lane 1) and no band in original strain (lane 2). Lane M indicates the DNA molecular weight marker (λ-*Eco*T14I digest). **(c)** PCR analysis with the genomic DNA from original strain and *S. kanamyceticus* JKE2, using primers R13JK3 and R13JK4 (indicated in (a)); 3641bp corresponding to intact *kanJ* and *kanK* gene and R3 in *S. kanamyceticus* JKE2 (lane 1) and no band in original strain (lane 2). Lane M indicates the DNA molecular weight marker (λ-*Eco*T14I digest).
