# Supplementary material for: Modulation of kanamycin B and kanamycin A biosynthesis in Streptomyces kanamyceticus via metabolic engineering
Source: PLoS One. 2017 Jul 28;12(7):e0181971. doi: 10.1371/journal.pone.0181971 (PMC5533434; doi:10.1371/journal.pone.0181971)
Supplement: S8 Fig — (DOCX) [file pone.0181971.s010.docx]

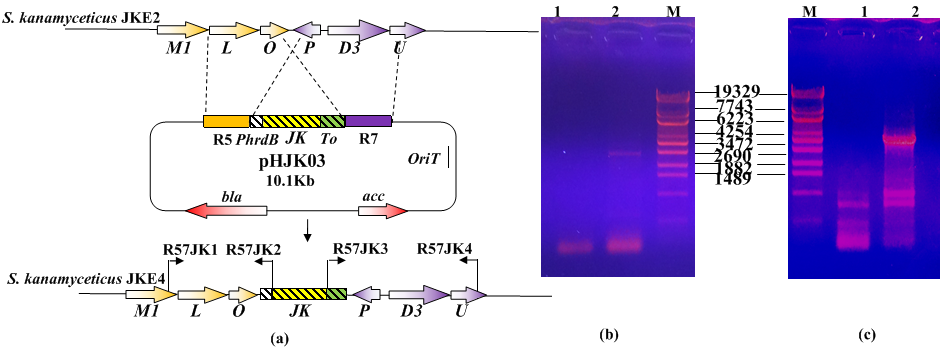
**S8 Fig. Construction of the *kanJ-* and *kanK-*overexpressing strain *S. kanamyceticus* JKE4.**

**(a)** Genotype of mutant strain *S. kanamyceticus* JKE2 and *S. kanamyceticus* JKE4. **(b)** PCR analysis with the genomic DNA from *S. kanamyceticus* JKE2 and *S. kanamyceticus* JKE4, using primers R57JK1 and R57JK2 (indicated in (a)); 2526bp band corresponding to intact R5 and *PhrdB* promoter in *S. kanamyceticus* JKE4 (lane 2) and no band in *S. kanamyceticus* JKE2 (lane 1). Lane M indicates the DNA molecular weight marker (λ-*Eco*T14I digest). **(c)** PCR analysis with the genomic DNA from *S. kanamyceticus* JKE4 and *S. kanamyceticus* JKE2, using primers R57JK3 and R57JK4 (indicated in (a)); 3610bp band corresponding to intact *To* terminator and R7 in *S. kanamyceticus* JKE4 (lane 2) and no band in *S. kanamyceticus* JKE2 (lane 1). Lane M indicates the DNA molecular weight marker (λ-*Eco*T14I digest).
