# Supplementary material for: Modulation of kanamycin B and kanamycin A biosynthesis in Streptomyces kanamyceticus via metabolic engineering
Source: PLoS One. 2017 Jul 28;12(7):e0181971. doi: 10.1371/journal.pone.0181971 (PMC5533434; doi:10.1371/journal.pone.0181971)
Supplement: S9 Fig — (DOCX) [file pone.0181971.s011.docx]

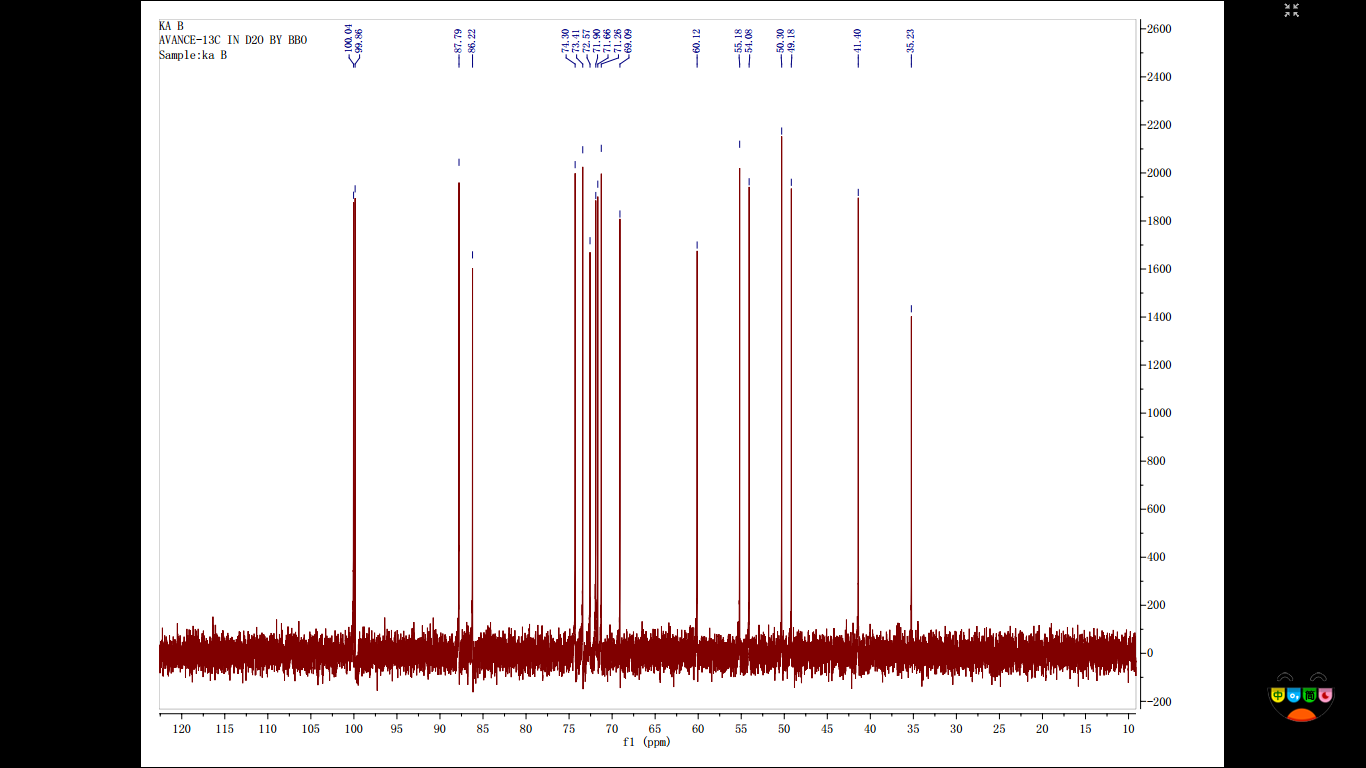
**S9 Fig** **^13^C NMR spectrum of the** [**main**](C:/Users/Administrator/AppData/Local/Yodao/DeskDict/frame/20160219111515/javascript:void(0);) [**products**](C:/Users/Administrator/AppData/Local/Yodao/DeskDict/frame/20160219111515/javascript:void(0);) **of *S. kanamyceticus* Δ*kanJ***
